# Evaluation of Anthropometric and Metabolic Parameters in Obstructive Sleep Apnea

**DOI:** 10.1155/2015/189761

**Published:** 2015-07-14

**Authors:** Yaşar Yildirim, Süreyya Yilmaz, Mehmet Güven, Faruk Kılınç, Ali Veysel Kara, Zülfükar Yilmaz, Gökhan Kırbaş, Alpaslan Kemal Tuzcu, Fatma Yılmaz Aydın

**Affiliations:** ^1^Department of Internal Medicine, Faculty of Medicine, Dicle University, Diyarbakır, Turkey; ^2^Department of Pulmonology, Faculty of Medicine, Dicle University, Diyarbakır, Turkey

## Abstract

*Aims*. Sleep disorders have recently become a significant public health problem worldwide and have deleterious health consequences. Obstructive sleep apnea (OSA) is the most common type of sleep-related breathing disorders. We aimed to evaluate anthropometric measurements, glucose metabolism, and cortisol levels in patients with obstructive sleep apnea (OSA). *Materials and Methods*. A total of 50 patients with a body mass index ≥30 and major OSA symptoms were included in this study. Anthropometric measurements of the patients were recorded and blood samples were drawn for laboratory analysis. A 24-hour urine sample was also collected from each subject for measurement of 24-hour cortisol excretion. Patients were divided equally into 2 groups according to polysomnography results: control group with an apnea-hypopnea index (AHI) <5 (*n* = 25) and OSA group with an AHI ≥5 (*n* = 25). *Results*. Neck and waist circumference, fasting plasma glucose, HbA1c, late-night serum cortisol, morning serum cortisol after 1 mg dexamethasone suppression test, and 24-hour urinary cortisol levels were significantly higher in OSA patients compared to control subjects. Newly diagnosed DM was more frequent in patients with OSA than control subjects (32% versus 8%, *p* = 0.034). There was a significant positive correlation between AHI and neck circumference, glucose, and late-night serum cortisol. *Conclusions*. Our study indicates that increased waist and neck circumferences constitute a risk for OSA regardless of obesity status. In addition, OSA has adverse effects on endocrine function and glucose metabolism.

## 1. Background

Obstructive sleep apnea (OSA) is the most common type of sleep-related breathing disorders. This disorder is characterized by excessive daytime sleepiness, snoring, and episodes of apnea. Cardiovascular diseases, which are among long-term adverse effects of OSA, lead to increased mortality and morbidity [1]. The only relevant study from our country reported an OSA prevalence of 0.9–1.9% [2]. The prevalence of the disease peaks between 40 and 65 years of age and decreases after 65 years of age [3, 4]. In fact, 25% of men aged 30 to 60 years meet minimal criteria (apnea-hypopnea index greater than 5) of OSA [5]. The gold standard test for diagnosis is polysomnography (PSG).

In order to sustain life, the hypothalamus-pituitary-adrenal (HPA) axis should function to elicit the normal cortisol response to stress. In stress-free states the normal cortisol release shows a diurnal pattern; that is, the highest cortisol levels are observed early in the morning (04:00–08:00), while the lowest levels occur late at night (02:00–04:00). Patients with OSA frequently exhibit increased ventilatory effort, sleep fragmentation, and oxygen desaturation. These events produce stress in an individual's body, activating the HPA axis that increases cortisol production. Cortisol has many functions including regulation of blood glucose, vascular tone, and myocardial contractility.

OSA increases the risk of hypertension, diabetes mellitus (DM), myocardial infarction, congestive heart failure, and stroke [6]. In our study, our goal was to evaluate anthropometric measurements, glucose metabolism, and cortisol levels in patients with OSA.

## 2. Materials and Methods

### 2.1. Study Population

A total of 50 patients with a body mass index ≥30 and major OSA symptoms including snoring, daytime sleepiness, and witnessed apnea were included in this study. We conducted this study between January 2012 and September 2013 at Department of Endocrinology, Faculty of Medicine, Dicle University. We obtained approval from a local medical research ethics committee. All the following types of patients were excluded from our study: patients with previous diagnosis of OSA, continuous positive airway pressure (CPAP) treatment, or bilevel positive airway pressure (BiPAP) therapy, patients with type 2 DM, hypertension, hypothyroidism, Cushing syndrome, hyperaldosteronism, pregnancy, malignancy, congestive heart failure, psychotic disorder, chronic liver failure, nephrotic syndrome, or chronic renal failure, or patients using drugs that had a potential to impair study tests (angiotensin converting enzyme inhibitor, angiotensin receptor blocker, phenytoin, barbiturate, or oral contraceptive). All subjects entering the study gave a written informed consent prior to the study, and all study tests and examinations were performed in compliance with the Helsinki Declaration regulating biomedical research on humans.

### 2.2. Study Procedures

Blood samples for fasting glucose, insulin, HbA1c, adrenocorticotropic hormone (ACTH), and cortisol were drawn at 08:00 from each patient during their stay in the endocrinology department. Newly diagnosed diabetes mellitus was determined according to ADA criteria [7]. Homeostatic model assessment insulin resistance (HOMA-IR), a method used to quantify insulin resistance and beta-cell function, was measured as glucose × insulin/405. A 24-hour urine sample was also collected from each subject for measurement of 24-hour cortisol excretion.

An additional blood sample was drawn for cortisol level at 23:00. A 1 mg dexamethasone suppression test was performed on the next day. A 1 mg decort tablet was administered at 23:00 and cortisol level was measured at 08:00 the next morning. A cortisol level less than 1.8 *μ*g/dL (50 nmol/L) suggested a suppressed cortisol level. A low dose dexamethasone suppression test with 2 mg dexamethasone was performed for 2 days in patients with high cortisol levels.

Body weight, length, waist circumference, and neck circumference were measured with a nonflexible tape and recorded in centimeters. Body mass index was calculated with the formula of body weight/height^2^ (kg/m^2^). Waist circumference was measured from the diameter between the costal arch and the anterior superior iliac spine at umbilicus level. Neck circumference was measured from the level of superior border of cricothyroid membrane. The same person performed all the measurements using the same tools; the results were rounded to the closest 0.5 cm to minimize measurement errors.

After completion of the procedures, each patient had a polysomnographic recording at Dicle University, Department of Chest Diseases, Center of Sleep Disorders. The polysomnographic recordings were carried out using a 32-channel E series polysomnography device (Compumedics E Series, Compumedics Limited, Abbotsford, VIC, Australia). The patients were monitored for at least 8 hours during sleep. Electroencephalography (EEG) recordings were made from 4 channels designated as C3/A2, C4/A1, O1/A2, O2/A1 according to the International 10–20 system that determines the locations of the electrodes. Left-right electrooculography (EOG), mental electromyography (EMG), and electrocardiography (ECG) recordings were also performed.

A transducer system connected to a nasal cannula was used to record respiratory events. The respiratory movements were recorded with the help of thoracic and abdominal tapes. Snoring was monitored with a microphone placed on the larynx at the anterosuperior portion of the neck. Oxygen saturation (SO_2_) was measured with a pulse oximetry during sleep. Leg movements were recorded with a bilateral pretibial EMG. PSG recordings were scored according to the Rechtschaffen and Kales criteria as 30-second epochs. All recordings were scored manually in a digital format by a physician certified in sleep disordered breathing. Sleep stages, respiratory events, and their characteristics were recorded. Hypopnea was defined as a reduction in nasal pressure signal of ≥50% that lasted ≥10 s, resulting in a ≥3% decrease in oxygen saturation from the prevent baseline or an arousal.

The control group in this study was comprised of patients who snored and had episodes of witnessed apnea and excessive daytime sleepiness and an AHI of <5 on polysomnography. The OSA group was composed of the study subjects who snored and had a witnessed apneic episode and excessive daytime sleepiness and an AHI equal to or greater than 5.

### 2.3. Statistical Analysis

Data analyses were performed using Statistical Package for Social Sciences (SPSS), Version 18.0 for Windows (SPSS Inc., Chicago, IL, USA). Normally distributed variables were presented using means and standard deviations. Student's* t*-test was used to compare the means of the continuous variables with normal distribution for related and independent samples. The chi-square test was used to compare these proportions in different groups. The Pearson correlation was used for simple regression analysis.* P* values less than 0.05 were considered statistically significant.

## 3. Results

A total of 50 patients were enrolled in the study and patients were divided equally into 2 groups according to polysomnography results: control group (*n* = 25) and OSA group (*n* = 25). The mean age was 43.76 ± 8.36 year and the male-to-female ratio was 13 : 12 in the control group. In the OSA group, the mean age was 46.68 ± 5.75 years with a male-to-female ratio of 12 : 13. There were no differences between groups for age and gender distribution (*p* > 0.05).

**Figure 1:**
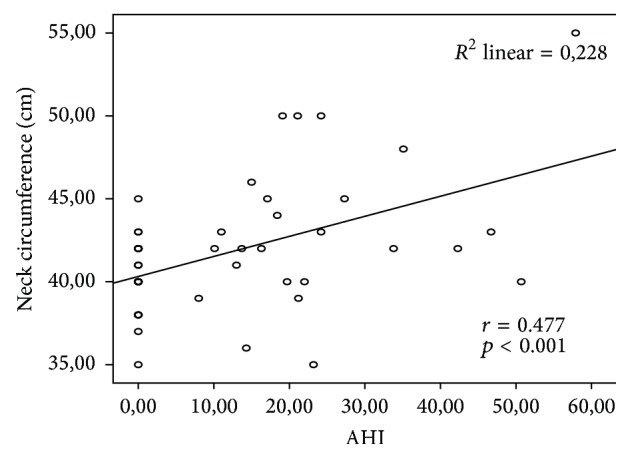
Pearson correlation analysis between AHI and neck circumference.

When comparing anthropometric measurements between the control group and OSA group, neck circumference (43.28 ± 4.62 cm versus 40.28 ± 2.17 cm, *p* < 0.01) and waist circumference (131.44 ± 20.86 cm versus 116.28 ± 23.5 cm, *p* < 0.05) were significantly higher in OSA group compared to control group. Body mass index was not significantly different between the groups (46.91 ± 12.86 kg/m^2^ versus 39.87 ± 13.36 kg/m^2^, *p* > 0.05). Demographic characteristics and anthropometric measurements of the groups are presented in Table 1. There was a significant positive correlation between AHI and neck circumference (*r* = 0.477, *p* < 0.001; Figure 1).

**Figure 2:**
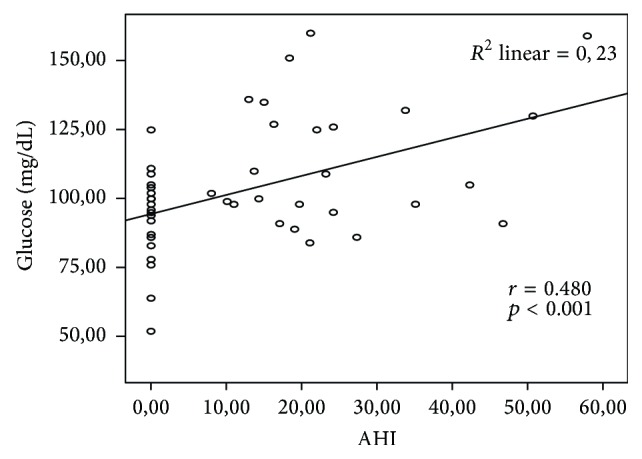
Pearson correlation analysis between AHI and glucose.

Compared to control subjects, OSA patients had significantly higher levels of fasting plasma glucose (113.44 ± 22.93 mg/dL versus 92.24 ± 15.7 mg/dL, *p* < 0.01) and HbA1c (6.80 ± 0.90% versus 5.7 ± 0.60%, *p* < 0.01). There was no significant difference between groups in insulin levels and homeostatic model assessment insulin resistance (HOMA-IR). Newly diagnosed DM was more frequent in patients with OSA than control subjects (32% versus 8%, *p* = 0.034). Results are shown in Table 2. There was a significant positive correlation between AHI and plasma glucose (*r* = 0.480, *p* < 0.01; Figure 2).

**Figure 3:**
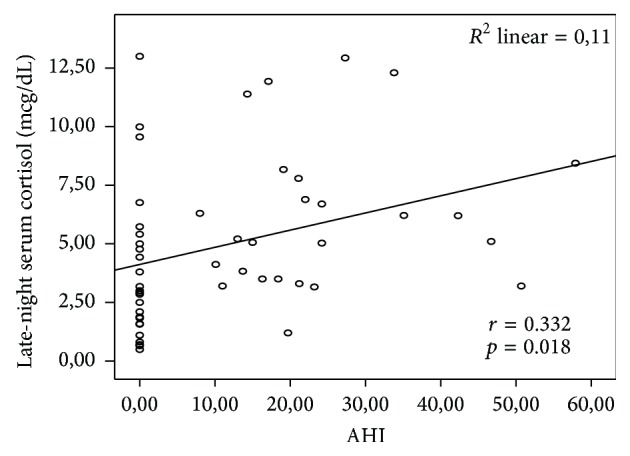
Pearson correlation analysis between AHI and late-night serum cortisol.

As shown in Table 3, late-night serum cortisol (6.18 ± 3.19 mcg/dL versus 3.82 ± 3.19 mcg/dL, *p* < 0.05), morning serum cortisol after 1 mg dexamethasone suppression test (1.53 ± 1.09 mcg/dL versus 0.87 ± 0.45 mcg/dL, *p* < 0.01) and 24-hour urinary cortisol (81.96 ± 68.04 *μ*gr/day versus 44.33 ± 42.07 *μ*gr/day, *p* < 0.05) levels were significantly higher in the OSA group than control group. However, morning serum cortisol and adrenocorticotropic hormone (ACTH) levels were not significantly different between the groups. There was a significant positive correlation between AHI and late-night serum cortisol (*r* = 0.332, *p* = 0.018, Figure 3).

A 1 mg dexamethasone suppression test was administered to all patients. Plasma cortisol levels were not suppressed in 1 of 25 patients (4%) in the control group and in 5 out of 25 patients (20%) in the OSA group (*p* = 0.189). When a 2 mg dexamethasone suppression test was applied, plasma cortisol levels were suppressed in both group.

## 4. Discussion

Obstructive sleep apnea is a syndrome characterized by snoring, excessive daytime sleepiness, and oxygen desaturation as a result of repeated upper airway collapse during sleep.

The prevalence of this disorder increases with age and peaks between ages 40 and 65. The male-to-female ratio has reportedly been in the range of 2 : 1-3 : 1 for premenopausal women and 1 : 1 for postmenopausal woman. In our study the mean age of the OSA group was 46.68 ± 5.71 years. The male-to-female ratio was 1 : 1 due to relatively large proportion of postmenopausal women in the present study.

Obesity causes an increased tendency for OSA. As a general rule, obese OSA patients have a bigger tongue and a narrower upper airway passage. In addition, obese OSA patients have also diminished respiratory muscle strength [8]. Obesity reduces total respiratory compliance by decreasing both chest wall compliance and lung compliance. These combined effects lead to a decrease in functional residual capacity, vital capacity, and total lung capacity as well as an increased airway resistance. Abdominal obesity can reduce lung volume particularly in supine position and may reflexively affect upper airway dimensions. When lung volume regresses from total lung capacity to residual volume, the pharyngeal cross-sectional area is reduced and pharyngeal resistance increases [9].

Therefore, it is possible that obesity increases susceptibility to OSA. The risk for OSA increases 8–12 times in persons with a BMI greater than 28 [10]. This risk further increases in persons with upper body obesity and those with a BMI > 40 [11]. Neck circumference reflects upper body obesity and is considered to be a better marker than BMI for OSA [12]. A neck circumference greater than 43 cm in men and 38 cm in women increases the risk of OSA [13]. In our study, 11 out of 13 women had a neck circumference greater than 38 cm. Seven of 12 men had a neck circumference greater than 43 cm. All patients in our study had obesity (BMI > 30). Additionally, we observed that neck circumference and waist circumference were significantly higher in the OSA group compared to control group, while body mass index was not significantly different between the groups. In addition, there was a significant positive correlation between AHI and neck circumference (*r* = 0.477, *p* < 0.001). This finding supported the idea that neck circumference alone is a risk factor for OSA independent of obesity.

Repetitive respiratory difficulties during sleep cause intermittent hypoxia and alterations in intrathoracic pressure that trigger autonomic responses. Among these autonomic alterations, increased sympathetic activity plays an important role. Increased sympathetic activity, sleep fragmentation, and intermittent hypoxia contribute to metabolic dysfunction [14, 15]. Specifically, efficiency of glucose transport and insulin resistance have been linked to metabolic dysfunction. Cortisol decreases insulin sensitivity and adds to glucose intolerance at all hours of a day. Sleep deprivation itself causes HPA axis hyperactivity and negatively affects glucose tolerance [16].

In a 270-patient study, Ip et al. [17] grouped 185 patients with OSA into obese and nonobese groups and compared the two groups. They found a significant elevation in fasting insulin levels and HOMA-IR values in the OSA group with an AHI greater than 5, while they could not detect any significant difference between obese and nonobese groups in terms of insulin level and insulin resistance. We found no significant difference between the two groups with respect to HOMA-IR values that reflect insulin resistance. This may have been due to the small sample size. However, OSA patients had significantly higher levels of plasma glucose and HbA1c compared to control subjects. In addition, in the present study newly diagnosed DM was more frequent in patients with OSA than control subjects (32% versus 8%, *p* = 0.034). As patients with OSA are usually obese, their risk of having or developing insulin resistance, type 2 DM, and hypertension is increased. Nocturnal cortisol elevations in these patients further increase the already heightened risk. The prevalence of type 2 DM is around 30% in patients with OSA [18].

Cortisol release shows a diurnal variation in normal people. The HPA axis secretes cortisol in a pulsatile manner at morning hours and sets cortisol to release into blood stream at a minimum level at night. That is, the highest cortisol levels are observed early in the morning (04:00–08:00) while the lowest ones occur late at night (02:00–04:00). Daily cortisol production is at a level of 5.7 mg/m^2^/day [19]. An estimated daily cortisol production of around 10–17 mg takes place in the normal healthy population and varies depending on body habitus, age, and sex [20]. In stressful conditions cortisol has a vital role for survival of an organism. It has been observed that the diurnal rhythm of cortisol is impaired in sleep disorders, certain acute and chronic abnormalities (infections, drugs, inflammatory disease, etc.), and conditions such as Cushing syndrome [21, 22]. Sleep deprivation leads to pulsatile cortisol release together with sympathetic activation in patients with OSA [23]. Sympathetic activation induces catecholamine release and especially corticotropin releasing hormone and cortisol secretion.

Various studies found no difference between OSA patients and the control group with respect to morning cortisol levels, although nocturnal cortisol levels have been found to be higher in OSA patients [24–26]. Grunstein et al. compared plasma cortisol levels at 06:00 between OSA and control groups and found no significant difference [27]. Lanfranco et al. found similar morning plasma ACTH, cortisol, and 24-hour urinary free cortisol levels in obese and nonobese OSA patients and control group [28]. We found no significant difference between the two groups with regard to morning cortisol level although nocturnal cortisol level was significantly higher in OSA group (*p* < 0.05). The severity of OSA worsens as the AHI increases. Thus, frequent awakenings and duration of hypoxia also increase. Also in our patients an increase in AHI was correlated with a nocturnal cortisol elevation (*r* = 0.332, *p* = 0.018), suggesting that stress and night wakefulness boost the activity of HPA axis and increase pulsatile cortisol release.

All of our patients underwent a 1 mg dexamethasone suppression test. The cortisol level was 0.87 ± 0.45 mcg/dL in the control group and 1.53 ± 1.09 mcg/dL in the OSA group (*p* < 0.01). This suggests that it is more difficult for OSA patients to suppress cortisol with a 1 mg dexamethasone suppression test. Patients with OSA had a higher 24-hour urinary cortisol level compared to the control group. These two findings support nocturnal cortisol elevation in patients with OSA which is in line with the two previous studies [24, 29].

Sleep disorders have recently become a significant public health problem both in our country and worldwide. As the number of people with obesity and obesity-related diseases increases, the number of patients suffering from OSA increases. Obesity, along with increased neck and waist circumferences, increases the risk of OSA. Hypoxemia and stress induced by OSA further worsen the impact of obesity on metabolic and endocrine systems.

## 5. Conclusions

This study reveals that recognition of newly diagnosed type 2 diabetes mellitus in 32% of OSA patients and especially increased nocturnal cortisol levels in OSA patients both point to an adverse effect of OSA on the endocrine system. One additional significant finding of our study was that increased waist and neck circumferences constituted a risk for OSA regardless of obesity status. We suggest that endocrinological and anthropometric examinations are necessary for OSAS patients. We hope that the current study will contribute significantly to the literature. A limitation of the study was the relatively small sample size; therefore further division of OSAS group could not be examined. We also believe that further studies with larger sample size are needed on this subject.

## Figures and Tables

**Table 1:** Demographic characteristics and anthropometric measurements in control group versus OSA group.

Parameters	Control group (*n* = 25)	OSA group (*n* = 25)	*p*
Age (years)	43.76 ± 8.36	46.68 ± 5.75	>0.05
Gender (M/F)	13/12	12/13	>0.05
Height (cm)	164.64 ± 9.40	166.56 ± 8.76	>0.05
Weight (kg)	106.22 ± 29.20	129.72 ± 35.80	**<0.05**
BMI (kg/m^2^)	39.87 ± 13.36	46.91 ± 12.86	>0.05
Neck circumference (cm)	40.28 ± 2.17	43.28 ± 4.62	**<0.01**
Waist circumference (cm)	116.28 ± 23.5	131.44 ± 20.86	**<0.05**
AHI	2.25 ± 0.48	24.21 ± 6.75	**<0.001**

BMI: body mass index.

**Table 2:** Glucose metabolism in control group versus OSA group.

Parameters	Control group (*n* = 25)	OSA group (*n* = 25)	*p*
Fasting glucose (mg/dL)	92.24 ± 15.17	113.44 ± 22.93	**<0.01**
Insulin (Uu/mL)	33.76 ± 34.07	36.72 ± 34.74	>0.05
HbA1c (%)	5.7 ± 0.60	6.80 ± 0.90	**<0.01**
HOMA-IR	7.95 ± 8.08	10.67 ± 10.83	>0.05
Newly diagnosed DM	8%	32%	**0.034**

HbA1c: hemoglobin A1c; HOMA-IR: homeostatic model assessment insulin resistance.

**Table 3:** ACTH and cortisol levels in control group versus OSA group.

Parameters	Control group (*n* = 25)	OSA group (*n* = 25)	*p*
Morning serum ACTH (pg/mL)	35.56 ± 20.82	36.72 ± 21.61	>0.05
Morning serum cortisol (mcg/dL)	12.47 ± 6.25	12.05 ± 4.57	>0.05
Late-night serum cortisol (mcg/dL)	3.82 ± 3.19	6.18 ± 3.19	**<0.05**
24-hour urinary cortisol (*μ*gr/day)	44.33 ± 42.07	81.96 ± 68.04	**<0.05**
Morning serum cortisol after 1 mg dexamethasone suppression test (mcg/dL)	0.87 ± 0.45	1.53 ± 1.09	**<0.01**
